# COVID-19 conspiracy ideation is associated with the delusion proneness trait and resistance to update of beliefs

**DOI:** 10.1038/s41598-022-14071-7

**Published:** 2022-06-20

**Authors:** K. Acar, O. Horntvedt, A. Cabrera, A. Olsson, M. Ingvar, A. V. Lebedev, P. Petrovic

**Affiliations:** Department of Clinical Neuroscience, Karolinska Institutet, K8 Klinisk neurovetenskap, K8 Neuro Ingvar, 171 77 Stockholm, Sweden

**Keywords:** Psychology, Human behaviour, Personality, Psychosis, Schizophrenia

## Abstract

The rapid spread of conspiracy ideas associated with the recent COVID-19 pandemic represents a major threat to the ongoing and coming vaccination programs. Yet, the cognitive factors underlying the pandemic-related conspiracy beliefs are not well described. We hypothesized that such cognitive style is driven by delusion proneness, a trait phenotype associated with formation of delusion-like beliefs that exists on a continuum in the normal population. To probe this hypothesis, we developed a COVID-19 conspiracy questionnaire (CCQ) and assessed 577 subjects online. Their responses clustered into three factors that included Conspiracy, Distrust and Fear/Action as identified using principal component analysis. We then showed that CCQ (in particular the Conspiracy and Distrust factors) related both to general delusion proneness assessed with Peter’s Delusion Inventory (PDI) as well as resistance to belief update using a Bias Against Disconfirmatory Evidence (BADE) task. Further, linear regression and pathway analyses suggested a specific contribution of BADE to CCQ not directly explained by PDI. Importantly, the main results remained significant when using a truncated version of the PDI where questions on paranoia were removed (in order to avoid circular evidence), and when adjusting for ADHD- and autistic traits (that are known to be substantially related to delusion proneness). Altogether, our results strongly suggest that pandemic-related conspiracy ideation is associated with delusion proneness trait phenotype.

## Introduction

During the COVID-19 vaccination circulating conspiracy theories have posed a serious threat to fully achieve the goal of the vaccination program^1–3^. Freeman et al. estimated that while 25% showed a degree of endorsement, 15% showed a consistent pattern of endorsement, and 10% had very high levels of endorsement for such ideas in an online survey with 2501 adults in England^4^. Such false beliefs likely contribute significantly to why as many as 25% of US citizens may refuse vaccination^5,6^. Yet, the cognitive factors and underlying mechanisms the pandemic-related conspiracy beliefs are not well described.

It has been suggested that contextual psychological factors such as social influences (such as peer pressure between proximate networks)^7–9^ and framing of information^8,10^ are particularly relevant for conspiracy ideas surrounding COVID-19 pandemic in the population on a group level^1,2^. However, emphasis on specific psychological contextual factors, assuming that anyone can equally believe in COVID-19 conspiracy ideas, does not fully account for the phenomenon why such beliefs exist in large percentage of the population. Likewise, specific pathological conditions^3^ cannot explain the high prevalence of these phenomena in the general population.

A factor that may explain inter-individual variability in the propensity for such beliefs is the delusion proneness trait. This trait phenotype is associated with formation of delusion-like beliefs and exists on a continuum in the population (i.e. low to high degree of delusion proneness)^11^, which have displayed a good test–retest reliability up to one year later^12^, indicating that it is relatively stable over time. Individuals who score high on delusion proneness shows cognitive, thought- and perceptual characteristics similar to the ones seen in psychosis-spectrum disorders, but on a non-clinical level^11,13–16^. It has been suggested that such phenotypes integrate explicit information to a larger degree than others in order to compensate for a more noisy and unprecise low level information processing^14,17,18^, thus making higher order priors more precise in a predictive coding frame work^18^. According to this model, when a novel belief has been adopted in such phenotypes, it is harder to change it, even in face of new evidence. This cognitive aberration is elegantly captured in Bias Against Disconfirmatory Evidence (BADE) tasks^13^, which has reliably been shown in several psychosis-related conditions and traits including schizophrenia patients^19–23^, individuals with at-risk-mental-states for psychosis^24^, subjects with schizotypy^25,26^ and in healthy subjects scoring high on delusion proneness^27,28^.

Recent studies have suggested that paranoia^29^ and delusion proneness traits^30^ may drive COVID-19 conspiracy ideas. Studies based only on ratings, such as Larsen et al.^30^, are limited in their interpretation since they are subjective by nature. Kuhn et al. also performed a series of objective cognitive tests, including an online BADE version, suggesting differences in information processing style in subjects adhering to conspiracy ideas^29^. This underlies the notion that conspiracy ideation is related to a specific phenotype. Although, meticulously designed, the study by Kuhn et al. has a limitation of not fully capturing the delusion proneness trait, instead focusing on paranoia that specifically characterizes mistrust and persecutory beliefs, whereas delusion proneness represents a broader construct associated with e.g., grandiosity, paranormal beliefs, thought disturbances etc.^12^.

Finally, we previously have demonstrated a weak and moderate correlation between delusion proneness and autistic- and ADHD traits^31^ respectively, such factors may confound the results and is therefore important to take into account in order to disentangle the cognitive mechanisms of these ideas and beliefs.

In the present study, we wanted to address the limitations stated above. We first constructed the COVID-19 Conspiracy Questionnaire (CCQ) consisting of common COVID-19 conspiracy-related statements and examined it in relation to measures of delusion proneness and general conspiracy ideation in a sample of social media users. We also examined their evidence integration abilities using an online version of the BADE task, thus also relying on experimental data. Finally, we performed analyses that tried to provide broader characteristic of the links between unconventional cognitive styles and conspiracy ideation whilst adjusting the effects for other trats like ADHD and autism^31^ that have been shown to correlate with delusion proneness.

## Methods

### Subject and general study design

We invited 1032 subjects that previously had completed a screening survey to fill out our COVID-19 Conspiracy Questionnaire (CCQ; further described in Supplements) and complete a Bias Against Disconfirmatory Evidence (BADE) task^13^ online. All subjects gave informed consent prior to participation. 616 subjects answered the CCQ questionnaire, and 577 of them had previously completed the screening survey, whereas a subsample of 313 also completed the online BADE (298 in Swedish and 15 in English). We had previously collected measures of delusion proneness using Peters Delusions Inventory (PDI), sex, age, education (how many years of University education or similar), as well as previous history of psychiatric diagnosis (major depressive disorder, psychotic disorder, obsessive compulsive disorder, autism spectrum disorder, attention deficit hyperactivity disorder, post-traumatic stress disorder and anxiety disorder).

The age range of our sample of 577 subjects was 15–67 years (*M* = 28.2, *SD* = 6.47), education range was 0–12 years (*M* = 2.89, *SD* = 2.55). The results of the questionnaires for this sample was: PDI (*M* = 6.06, *SD* = 4.19), CCQ Total score (*M* = 70.96, *SD* = 21.31), CCQ *Fear/Action* (*M* = 35, *SD* = 10.87), CCQ Distrust (*M* = 14.5, *SD* = 8.21), CCQ Conspiracy (*M* = 16.76, *SD* = 12.29).

For the subsample of 313 subjects that also completed the BADE the age range was 15–64 years (*M* = 28.4, *SD* = 6.62), range of education was 0–15 years (*M* = 3.19, *SD* = 2.64). The results of the questionnaires for this sample was: PDI (*M* = 5.97, *SD* = 4.15), CCQ Total score (*M* = 69.95, *SD* = 20.38), CCQ *Fear/Action* (*M* = 34.93, *SD* = 10.5), CCQ Distrust (*M* = 14.28, *SD* = 8.03), CCQ Conspiracy (*M* = 16.23, *SD* = 11.49).

Out of the subsample of subjects that completed BADE, 67 completed the PDI during 2018, 81 during 2019 and 165 during 2020. The BADE-task were completed from November 2020 to April 2021. All research was performed in accordance with relevant guidelines and regulations. The study was approved by the regional ethical board of Stockholm and the main outcome analyses were performed adhering to a preregistered protocol (https://osf.io/npb8q/).

### BADE task

The BADE task we used for this study was an online version of one introduced by Sanford and colleagues^32^. In this text-based version, subjects are presented with a statement accompanied with four different interpretations of the statement. Their task is to rate from a scale of 0 (very implausible) to 10 (very plausible) the plausibility of the different interpretations based on the statement. After their initial rating, an additional statement is added to the first one, and the subjects are given a chance to change their plausibility ratings according to the new information. Subjects receive three statements per scenario, and therefore give three plausibility ratings for each scenario. In total, there are 30 scenarios in this version. The BADE task is constructed in a way that for 24 of the scenarios, the true interpretation is only somewhat plausible at the beginning, but as more information is given, it becomes more and more obvious that it is the most plausible interpretation based on the statements. There are also two lure interpretations which initially seems plausible but becomes less plausible after new statements are given. Lastly, there is an absurd interpretation, which is highly implausible throughout the scenario. For 6 scenarios, the true interpretation is apparent after the first hint in order to prevent subjects from responding in a specific pattern. We scored Evidence Integration Impairment (EII) as proposed by Bronstein and Cannon, which is more parsimonious than previous methods, but still captures over 92% of the variance in previous metrics^33^.

### Quantification and statistical analysis

#### Data-normalization

As some of our variables were non-normally distributed, they were transformed using the “*bestNormalize*”^34^ package in *R*^35^*,* and were used for bivariate correlational analyses. As the residuals of our regression models were normally distributed, we did not use transformed variables when conducting the linear regression analyses.

#### Exploratory analyses

We also performed the main analyses after removing questions in PDI relating to paranoia (“Truncated PDI”; see Supplements). Finally, we performed the main analyses after adjusting for the effects of ADHD- and autistic traits assessed with Adult ADHD Self-Report Scale^36^) and self-rated autism symptoms (assessed with Ritvo Autism and Asperger Diagnostic Scale^37^) by adding the total scores as covariates to the model.

## Results

### Main analyses of the relation between CCQ, delusion proneness and BADE

**Figure 1:**
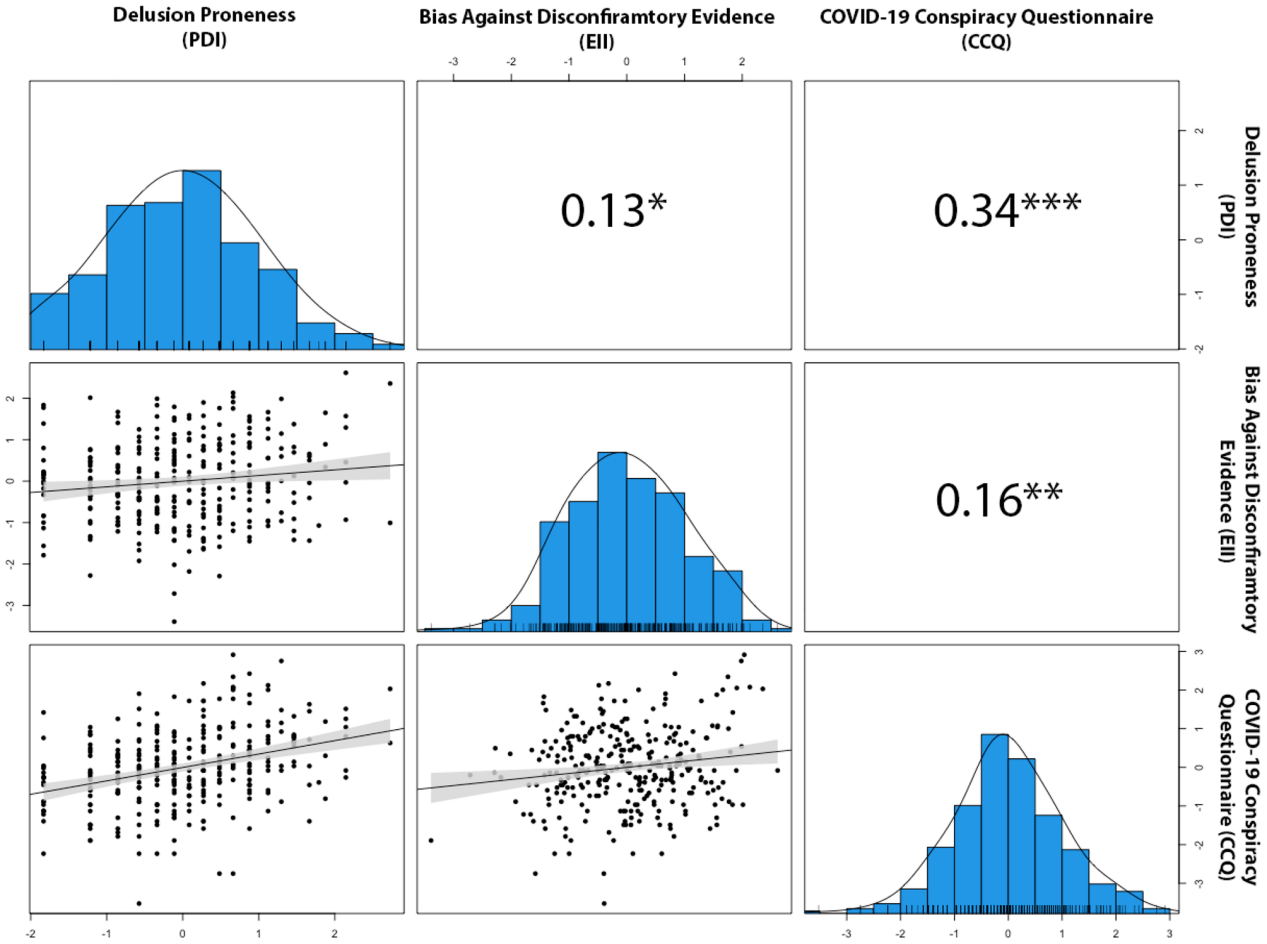
Pearson’s correlations with scatter and distribution plots of PDI, EII and CCQ total for subjects that completed BADE (N = 313); *p < 0.05, **p < 0.01, ***p < 0.001. All variables were transformed to achieve normality.

First, we showed that total score of CCQ was positively correlated with PDI (*N* = 577, *r* = 0.30, *p* < 0.001), with EII (*N* = 313, *r* = 0.16, *p* < 0.01) and with the Conspiracy Mentality Questionnaire (CMQ) which assess the general tendency for conspiracy ideation^42^ (*N* = 194, *r* = 0.53, *p* < 0.001). Note, that the first analysis remained significant in the smaller group that had completed BADE (Fig. 1).

We further performed a principal component analysis (PCA) to tease out different components contributing to conspiracy ideation. The PCA on the CCQ identified three factors; *Conspiracy*, *Distrust* and *Fear/Action* (see Supplements). While the *Conspiracy* factor incorporates classical conspiracy statements, the *Distrust* factor incorporates statements about distrust towards authorities and media, and the *Fear/Action* factor incorporates statements about general fear and avoidance behaviour—all in relation to the COVID-19 pandemic.

We then performed a series of multiple linear regression analyses to better understand the relation between CCQ and PDI (adjusting for the effect of age, sex, education and psychiatric diagnosis). The first model showed that total score of CCQ (*N* = 577, *t* = 7.58, *p* < 0.001), age (*t* = − 2.59, *p* < 0.01) and psychiatric diagnosis (*t* = 2.89, *p* < 0.005) predicted PDI. A second model, which included the CCQ components instead to the CCQ total points, showed that Conspiracy (*t* = 3.68, *p* < 0.001), Distrust (*t* = 4.34, *p* < 0.001), age (*t* = − 2.10, *p* < 0.05) and diagnosis (*t* = 2.76, *p* < 0.01) predicted PDI, whereas Fear/Action did not (*t* = 0.71, *p* = 0.48).

**Figure 2:**
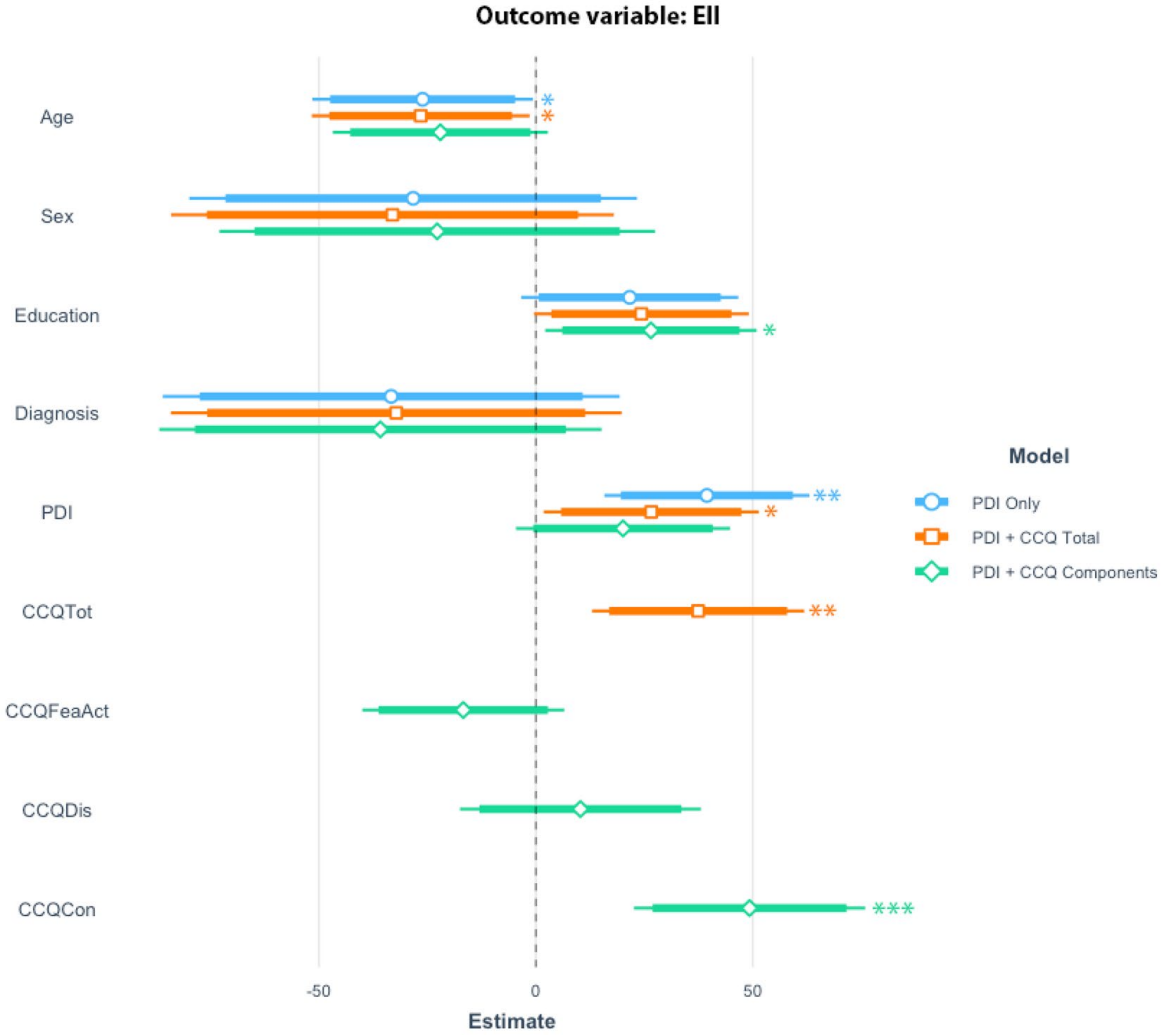
Regression coefficient plot for all three models with PDI (PDI Only), CCQ Total (PDI + CCQ Total) and CCQ components (PDI + CCQ Components) as predictors of EII (BADE). The figure shows the results of the different models, which gets increasingly complex, first model only shows only PDI whereas the second model shows the total CCQ-score, and the last and third model shows the score of the CCQ components, *p < 0.05, **p < 0.01, ***p < 0.001.

Next, regression models were performed to understand the relation between CCQ and BADE effect (indexed by EII) when also adjusting for PDI. A first model verified that PDI (*N* = 313, *t* = 3.28, *p* < 0.001), as well as age (*t* = − 2.02, *p* < 0.05), predicts EII. A second model which included CCQ suggested that PDI (*N* = 313, *t* = 2.11, *p* < 0.05), total CCQ (*t* = 3.01, *p* < 0.005), and age (*t* = − 2.08, *p* < 0.05) predicted EII. In the third model, in which CCQ was divided in its components, only the component Conspiracy (*t* = 3.64, *p* < 0.001), and education (*t* = 2.14, *p* < 0.05) predicted EII (see Fig. 2). In addition, we conducted principal component regression (PCR), sparse partial least squares regression (SPLS) as well as sparse principal component analysis (SPCA), which consistently showed that the *Conspiracy* factor was associated with EII (see Supplements for more details).

**Figure 3:**
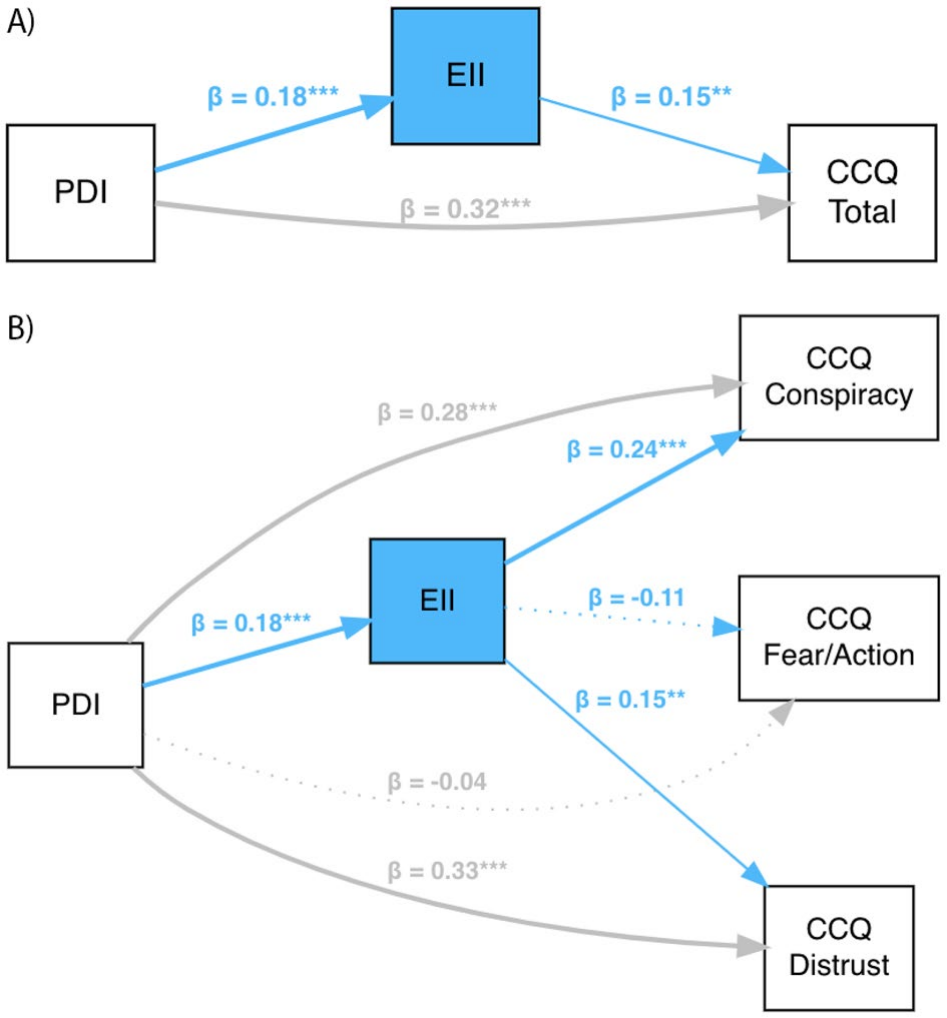
Results of path analyses with standardized parameter values. (**A**) Shows the CCQ Total path-model. (**B**)Shows the CCQ Components path-model; **p < 0.01, ***p < 0.001.

Lastly, we performed path analyses using *r* package (*lavaan)*^38^ using the Sobel test to examine the mediator role of EII between PDI and A) total CCQ and B) components of CCQ. The first model found that EII partially mediated the relationship between PDI and total CCQ (*β* = 0.027, *z* = 2.13, *p* < 0.05). The second model showed that EII partially mediated the relationship between PDI and the Conspiracy factor (*β* = 0.042, *z* = 2.61, *p* < 0.01), and between PDI and the Distrust factor (*β* = 0.026, *z* = 2.10, *p* < 0.05), but not between PDI and the Fear/Action factor (*β* = − 0.02, *z* = − 1.63, *p* = 0.10). See Fig. 3 and Supplements for further details.

### Exploratory analyses

#### Main results after removing paranoia relating questions in PDI (“truncated PDI)”

The significant correlation between delusion proneness and COVID-19 conspiracy ideation remained when using the truncated PDI version for the full CCQ (r = 0.309, p < 0.0001). Similarly, the truncated PDI version still showed a significant correlation with Conspiracy factor (r = 0.294, p < 0.0001) and the Distrust factor (r = 0.308 p < 0.0001) but not for the Fear/Action factor (r = − 0.0717, p = 0.084) for the full sample.

We then performed two regression analyses (as above) with the truncated PDI, adjusting for the effects of age, sex education and psychiatric diagnosis. The first model showed that the total score of CCQ (N = 577, t = 7.52, *p* < 0.001) and psychiatric diagnosis (*t* = 2.05, *p* < 0.05) predicted PDI. The second model which included CCQ components showed that Conspiracy (*t* = 3.66, *p* < 0.001) and Distrust (*t* = 4.17, *p* < 0.001) predicted PDI, whereas Fear/Action did not (*t* = 0.54, *p* = 0.59), showing similar results as the non-truncated PDI.

### Results after adjusting for ADHD and autistic traits

We assessed subclinical ADHD symptoms (with Adult ADHD Self-Report Scale^36^) and self-rated autism symptoms (with Ritvo Autism and Asperger Diagnostic Scale^37^), that we used for further adjustments in regression analyses. The relation between CCQ and PDI remained for the full CCQ scores (t = 6.702, p < 0.0001), as well as for the Conspiracy factor (t = 3.488, p < 0.001) and Distrust factor (t = 3.827, p < 0.0005) but not for the Fear/Action factor (t = 0.534 p = 0.5937) when replacing the full CCQ with the model that included the CCQ factors instead. See Supplements for detailed results.

After adjusting for ADHD- and autism traits, we also showed that the relation between CCQ and EII (BADE effect) remained significant for the model which included the full CCQ (t = 3.049, p < 0.005), as well as for the Conspiracy factor (t = 3.595, p < 0.0001) but not for the Fear/Action factor (t = − 1.211, p = 0.23) or Distrust factor (t = 0.679, p < 0.498) when replacing the full CCQ with the model which included CCQ factors. See Supplements for detailed results.

## Discussion

Our results show that COVID-19 conspiracy beliefs are related to the delusion proneness trait in regards to expressing general delusion associated ideas (measured with PDI). They also suggest an increased resistance to change of incorporated beliefs when novel evidence is presented (measured with BADE), often increased in delusion associated phenotypes^13^.

Importantly, the significant associations were found only for questions addressing COVID-19 related conspiracy ideas and distrust towards the authorities with respect to their reaction to the pandemic, but not general questions addressing COVID-19-related avoidance behaviour and fear. The latter were associated with delusion proneness and the BADE. Also, the relation between COVID-19 conspiracy beliefs and the BADE effect remained significant even when using the truncated PDI in which questions pertaining to paranoia were removed. Similarly, the path analyses suggested that PDI only partly mediated a direct effect on COVID-19 conspiracy beliefs, as another aspect was mediated through BADE. These results suggest that rated delusion proneness with PDI does not fully capture COVID-19 conspiracy beliefs, aspects that may be further explained by objective tests on information processing style.

Initial studies have suggested that there is a relation between delusion proneness and general conspiracy ideation^39,40^, not assessing specific delusional ideas or such beliefs related to the COVID-19 pandemic. More recently, a study has shown a relation between delusion proneness and COVID-19 conspiracy ideas^30^. Although indicative of a relation between delusion proneness and COVID-19 conspiracy ideas. Our results are also in line with a recent study^29^ showing that COVID-19 conspiracy ideas^4^ are also related to cognitive style (including BADE) and paranoia. Apart from BADE, this study used a set of objective tests focusing on jumping-to conclusions bias (JTC), liberal acceptance bias (LA), possibility of being mistaken (PM) and showing a different cognitive style and reasoning biases related to COVID-19 conspiracy ideas^29^. However, the study by Kuhn et al.^29^, although a large power and meticulously performed, does not fully capture the delusion proneness trait and has some interpretational limitations when assessing the underlying phenotype. The first limitation pertains to the use of paranoia, i.e. the assessed behavioral variable in the previous study^29^, which also is the main component of conspiracy ideation (including COVID-19 conspiracy beliefs). Second, delusion proneness is a multifaceted phenotype consisting of several different symptom-dimensions (and not only paranoia)^41^. Therefore, we used a validated delusion proneness questionnaire^12^ in our main analyses and a general conspiracy questionnaire^42^ only for validation purposes of CCQ. However, even when using the full delusion proneness trait in the analysis, paranoia symptoms may have driven the observed effects, also introducing a risk of circular evidence. In order to test that our results were not specifically related to paranoia, but to the delusion proneness trait phenotype, we performed our main analyses again after excluding questions pertaining to paranoia from the PDI. These analyses reproduced the main findings. Thus, his result points towards that it is not general paranoia that is related to COVID-19 conspiracy ideation but the full delusion proneness trait phenotype.

A third interpretational problem for both the previous study^29^ and our initial preregistered analyses is that the delusion proneness trait (including paranoia) is substantially correlated with ADHD-trait and autism-trait^31^ which may have confounded the results. In order to exclude these possible confounds, we performed similar regression analyses as presented in our main analyses, but that also included self-rated ADHD symptoms (assessed with Adult ADHD Self-Report Scale^36^) and self-rated autism symptoms (assessed with Ritvo Autism and Asperger Diagnostic Scale^37^). We found that the relation between CCQ and PDI remained for the full CCQ scores, as well as between PDI and the Conspiracy factor, and Distrust factor but not the Fear/Action factor (when replacing the full CCQ with the model that included the CCQ factors instead). Notably, both ADHD and autism traits also showed a significant relation with PDI (see Supplements) reproducing our previous findings^31^ and underlying the importance to adjust for those traits as well.

Similarly, after adjusting for ADHD- and autism traits, we showed that the relation between CCQ and EII (BADE effect) remained significant for the model which included the full CCQ, as well as for the Conspiracy factor—but not for the Fear/Action factor or Distrust factor (when replacing the full CCQ with the model which included CCQ factors). In sum, even after adjustments for several possible confounding traits our results still point towards that the delusion proneness phenotype is a main driver of COVID-19 conspiracy beliefs.

Notably, delusion proneness is a trait phenotype expressed in the non-clinical population, where people with high delusion proneness otherwise have normal function^11^. In light of this, the afore-mentioned characteristic should not be treated as pathological per se. Instead, this trait phenotype is associated with a range of specific behaviours that shape the personality of an individual, in a similar way as for the non-clinical range of ADHD and autism spectrum traits^43,44^. It is also likely that certain aspects of the delusion trait proneness (and associated phenotypes) in general and conspiracy beliefs in specific have a positive impact for the individual and society^45,46^. Nevertheless, the associated information processing has some similarities to the one observed in delusions^11,13–16^ that may be due to a shift in the balance between higher and lower order priors within a hierarchical predictive coding model^18^.

An important aspect of the present result is that it suggests that delusion proneness is not only associated with a resistance to change of beliefs (as experimentally captured in the BADE-task) but also to an increased propensity to incorporate novel beliefs that are not common (such as specific COVID-19 conspiracy ideas). At first, this seems as a paradox as it suggests both increased flexibility and increased inflexibility in belief formation. However, formation of new beliefs is in line with the aberrant salience and predictive coding theories of psychosis suggesting that such (trait-) phenotypes are associated with a noisy low-level processing of information and thus require compensatory increase in feedback signalling at higher levels of the hierarchy^17,18^. In line with these ideas, experimental research has shown sensory processing dysfunction^47^ suggesting noisy perceptual information processing. We have previously also shown that delusion proneness is associated with both noisy perceptual information processing and increased top-down effects after belief manipulation in an illusionary stimulus^14,48^.

Research on confirmation bias suggest that humans also tend to dismiss information that is not in line with their own beliefs^49,50^, an effect that seems to be stronger in psychosis related phenotypes^51^. However, this process is not only passive, as humans often actively seek for information that confirms their initial beliefs^52^. The combination of delusion proneness information processing style—that seem to include stronger initial belief formation, resistance to change those beliefs given novel evidence, as well as both increased passive and active confirmation bias—with belief bubbles and strengthened feedback from social media may be constitute a particularly malaise combination in terms of formation of novel conspiracy ideas, such as COVID-19 conspiracy beliefs, within the society.

Our study has a number of important limitations. First, most of the collected measures incorporated self-rating, limiting objectivity of assessment of the level of psychopathology. However, previous studies have shown a good test–retest reliability of PDI^12,53^, a scale specifically designed for self-rating. Apart from this, self-reported assessments of a related construct of has previously been shown to acceptably match the ones done by clinicians^54^. Another limitation is a cross-sectional nature of the employed assessments of delusion proneness, which does not capture the potential dynamics of the investigated trait. Thus, some of the investigated individuals may develop psychosis-spectrum disorders over the course of their lifetime (which will naturally lead to higher scores on the investigated variables), whilst others will remain stable on delusion proneness trait^11^. However, as mentioned above, previous studies have shown that PDI is a relatively stable measure with a good at least one-year test–retest reliability^12,53^. Finally, our study was specifically focused on COVID-19 related conspiracy thinking and the results do not necessarily generalize to other significant societal events. However, previous studies yielded similar associations of conspiracy ideation with schizotypy facets, delusion proneness and related cognitive styles, such as BADE^55,56^, thereby supporting generalizability of our findings.

Taken together, previous research^29^ and our results suggest that the cognitive style of information processing associated with delusion proneness in non-clinical populations is a major factor for driving conspiracy ideas related to the COVID-19 pandemic.

## Supplementary Information

Supplementary Information.

## Data Availability

The datasets and script for analysis for the current study are available in the GitHub repository, https://github.com/kasimacar/CCQOnlineBADE.
