# Supplementary material for: COVID-19 conspiracy ideation is associated with the delusion proneness trait and resistance to update of beliefs
Source: Sci Rep. 2022 Jun 20;12:10352. doi: 10.1038/s41598-022-14071-7 (PMC9208343; doi:10.1038/s41598-022-14071-7)
Supplement: Supplementary file 1 — Supplementary Information. [file 41598_2022_14071_MOESM1_ESM.docx]

**Supplements**

**COVID-19 conspiracy ideation is associated with the delusion proneness trait and resistance to update of beliefs**

Acar K, Horntvedt O, Cabrera A, Olsson A, Ingvar M, Lebedev AV*, Petrovic P*

Department of Clinical Neuroscience, Karolinska Institutet, Stockholm, Sweden

* = Contributed equally to the study

**Initial COVID-19** **Conspiracy Questionnaire (CCQ)**

1) I am trying to wash hands regularly to prevent the spread of the virus.

2) I am trying to avoid using public transport.

3) I am skeptical that any of the employed precautions will help to prevent or slow–down spread of the virus.

4) I am following the news in order to stay updated on recommended precautions regarding the coronavirus (SARS-CoV-2).

5) I am trying to stay at home avoiding contacts with other people.

6) The ongoing outbreak is a serious event, which, if not taken seriously, can lead to many deaths and other catastrophic consequences.

7)* The coronavirus (SARS-CoV-2) poses very little danger to my personal health.

8) Scientific conclusions about the coronavirus (SARS-CoV-2) are shaped by politics.

9) There is a vaccine or cure for coronavirus (SARS-CoV-2) that the government won't release.

10) The spread of the coronavirus (COVID-19) is a result of a secret initiative to handle the consequences of global warming.

11) Current situation around coronavirus (COVID–19) is a secret experiment to test how masses can be effectively controlled.

12) Official sources of information on COVID–19 cannot be trusted.

13) The magnitude and consequences of the new coronavirus (SARS-CoV-2) outbreak are deliberately twisted.

14) Progress toward a cure for the coronavirus is deliberately being hindered.

15) The rapid spread of the virus is the result of the deliberate, concealed efforts.

16) The real danger of the coronavirus is deliberately withheld from the public.

17) The ongoing outbreak is the result of a secret experiment on citizens.

18) Coronavirus (SARS-CoV-2) is a result of biochemical warfare.

19) SARS-CoV-2 was deliberately spread to target specific populations.

20) Current situation around coronavirus (COVID–19) was deliberately initiated to reduce global political tensions.

21) The spread of the coronavirus (SARS-CoV-2) is a sign from a higher power that wants to punish the human race.

22) The spread of the coronavirus (SARS-CoV-2) is the universe's/nature's way of stopping the global catastrophe.

* = reverse scoring

**Principal Component Analysis (PCA) of CCQ**

Two PCA were performed on the CCQ questions (which can be found below) with ‘varimax’ rotation. A threshold of >= 0.4 were used to identify significant loadings on components. In the first PCA, we identified three distinct components; *Conspiracy* (items 9-11, 14, 15 and 17-21); *Distrust* (items 3, 8, 12 and 13); and *Fear/Action* (items 1, 2, and 4-7). A second PCA was performed after removing item 16 and 22 due to significant cross-loadings on components *Conspiracy* and *Distrust* (delta <= 0.2) and no significant loading (< 0.4) on any component, respectively. The resulting component loadings and uniqueness from the second PCA are as follows: Component *Conspiracy* (Item 9: loading = 0.70, uniqueness = 0.44; Item 10: loading = 0.78, uniqueness = 0.37; Item 11: loading = 0.79, uniqueness = 0.32, Item 14: loading = 0.70, uniqueness = 0.47; Item 15: loading = 0.70, uniqueness = 0.41; Item 17: loading = 0.86, uniqueness = 0.23; Item 18: loading = 0.75, uniqueness = 0.41; Item 19: loading = 0.82, uniqueness = 0.31; Item 20: loading = 0.83, uniqueness = 0.29; Item 21: loading = 0.47, uniqueness = 0.78. Component *Fear/Action* (Item 1: loading = 0.49, uniqueness = 0.75; Item 2: loading = 0.68, uniqueness = 0.54; Item 4: loading = 0.57, uniqueness = 0.63; Item 5: loading = 0.77, uniqueness = 0.38; Item 6: loading = 0.68, uniqueness = 0.44; Item 7: loading = -0.56, uniqueness = 0.66). Component *Distrust* (Item 3: loading = 0.62, uniqueness = 0.59; Item 8: loading = 0.78, uniqueness = 0.37; Item 12: loading = 0.69, uniqueness = 0.41; Item 13: loading = 0.66, uniqueness = 0.36).

Component statistics are as follows: *Conspiracy*: SS Loading: 5.99, % of Variance: 29.96, Cumulative %: 29.96. *Fear/Action*: SS Loading: 2.50, % of Variance: 12.49, Cumulative % 42.45. *Distrust*: SS Loading: 2.35, % of Variance: 11.75, Cumulative % 54.20.

**Sparse Principal Component Analysis, Principal Component Regression and Sparse Partial Least Squares Regression**

In addition to conducting PCA and running regression analysis with the extracted components, we also conducted principal component regression (PCR), sparse partial least squares regression (sPLS) as well as sparse principal component analysis (sPCA).

**Sparse Principal Component Analysis (sPCA).** The sparse principal component analysis generated very similar components as the regular PCA; *Conspiracy* = items 9-11 and 14-21. The sparse PCA included item 16 in the Conspiracy component, while it was excluded in the PCA analysis. The *Fear/Action component* included items 2 and 4-6, while items 1 and 7 were excluded. The *Distrust* component included items 3, 8, 12-13, and 22, while item 22 was excluded in the PCA analysis. The loadings for the sparse PCA are as follows: Component *Conspiracy:* (Item 9: loading = 0.30, item 10: loading = 0.29, item 11: loading = 0.32, item 14: loading = 0.29, item 15: loading = 0.32, item 16: loading = 0.28, item 17: loading = 0.35, item 18: loading = 0.30, item 19: loading = 0.32, item 20: loading = 0.34, item 21: loading = 0.19). Component *Fear/Action: (*Item 2: loading = 0.51, item 4: loading =0.38, item 5: loading = 0.58, item 6: loading = 0.51)*.* Component *Distrust*: (Item 3: loading = 0.39, item 8: loading = 0.51, item 12: loading = 0.49, item 13: loading = 0.52, item 22: loading 0.27).

After extracting the components using sPCA, we conducted regression analyses similar to as in the main manuscript, which showed that the Conspiracy component (N = 313, *t* = 2.28, *p* = .023, Distrust (*t* = 4.57, *p* < .001) and psychiatric diagnosis (*t* = 2.25, *p* = .025) predicted PDI, while Fear/Action (*t* = 0.28, *p* = 0.78) did not. Our second regression analysis showed that the Conspiracy component (N = 313, *t* = 3.89, *p* < .001 and Education (*t* = 2.10 *p* = .037) predicted EII, while Distrust (*t* = 0.8, *p* = .42) and Fear/Action (*t* = -1.77, *p* = .078) did not predict EII.

**Principal Component Regression and Sparse Partial Least Squares Regression**

The results of the PCR yielded the following loadings: Item 3: loading = 0.13, item 8: loading = 0.19, item 9: loading = 0.27, item 10: loading = 0.26, item 11: loading = 0.29, item 12: loading = 0.22, item 13: loading = 0.25, item 14: loading = 0.26, item 15: loading = 0.28, item 16: loading = 0.26, item 17: loading = 0.31, item 18: loading = 0.27, item 19: loading = 0.28, item 20: loading = 0.30, item 21: loading = 0.17, item 22: loading = 0.11. Pearson’s correlation showed a nearly perfect correlation between the PCR scores and the *Conspiracy* component of the sPCA (*r* = .97, *t* = 74.4, *p* < .001). Similarly, the sPLS regression, showed a near-perfect correlation with the PCR scores (*r* = .99) as well as the *Conspiracy* component of the sPCA (*r* = .96).

**CCQ-questions sorted by the three factors:**

**Conspiracy**

9) There is a vaccine or cure for coronavirus (SARS-CoV-2) that the government won't release.

10) The spread of the coronavirus (COVID-19) is a result of a secret initiative to handle the consequences of global warming.

11) Current situation around coronavirus (COVID–19) is a secret experiment to test how masses can be effectively controlled.

14) Progress toward a cure for the coronavirus is deliberately being hindered.

15) The rapid spread of the virus is the result of the deliberate, concealed efforts.

17) The ongoing outbreak is the result of a secret experiment on citizens.

18) Coronavirus (SARS-CoV-2) is a result of biochemical warfare.

19) SARS-CoV-2 was deliberately spread to target specific populations.

20) Current situation around coronavirus (COVID–19) was deliberately initiated to reduce global political tensions.

21) The spread of the coronavirus (SARS-CoV-2) is a sign from a higher power that wants to punish the human race.

**Distrust**

3) I am skeptical that any of the employed precautions will help to prevent or slow–down spread of the virus.

8) Scientific conclusions about the coronavirus (SARS-CoV-2) are shaped by politics.

12) Official sources of information on COVID–19 cannot be trusted.

13) The magnitude and consequences of the new coronavirus (SARS-CoV-2) outbreak are deliberately twisted.

**Fear/Action**

1) I am trying to wash hands regularly to prevent the spread of the virus.

2) I am trying to avoid using public transport.

4) I am following the news in order to stay updated on recommended precautions regarding the coronavirus (SARS-CoV-2).

5) I am trying to stay at home avoiding contacts with other people.

6) The ongoing outbreak is a serious event, which, if not taken seriously, can lead to many deaths and other catastrophic consequences.

7)* The coronavirus (SARS-CoV-2) poses very little danger to my personal health.

* = reverse scoring

**Excluded Questions from the truncated version of Peter’s et al. Delusion Inventory**

**related to paranoia**

**Question 1:** Do you ever feel as if people seem to drop hints about you or say things with a double meaning?

**Question 4:** Do you ever feel as if you are being persecuted in some way?

**Question 5:** Do you ever feel as if there is a conspiracy against you?

**Question 13:** Are you often worried that your partner may be unfaithful?

**Question 15:** Do you ever feel that people look at you oddly because of your

appearance?

**Sample**

Of the sample from 577 subjects, 152 had a history of psychiatric diagnosis of which 6 had been diagnosed with schizophrenia, 45 with depression, 36 with bipolar disorder, 20 with ADHD, 24 with Autism, 13 with OCD, while 79 subjects also indicated “Other” which included PTSD, anxiety disorders and emotionally unstable personality disorder.

Of the subsample with 313 subjects, 85 had a history of psychiatric diagnosis of which 3 had been diagnosed with schizophrenia, 24 with depression, 15 with bipolar disorder, 15 with ADHD, 14 with Autism, 9 with OCD, while 32 subjects also indicated “Other” which included PTSD, anxiety disorders and emotionally unstable personality disorder.

**Detailed results of regression analyses controlling for ADHD and autistic traits**

**PDI - Total CCQ.** Regression analyses showed that total CCQ ( N = 577) *β* = 0.05, *t* = 6.70, *p* < .001), Adult ADHD Self-Report Scale (ASRS) (*β* = 0.12, *t* = 8.72, *p* < .001), Ritvo Autism Asperger Diagnostic Scale Revised (symptoms displayed while young) (RAADSY) (*β* = 0.25, *t* = 2.73, *p* < .01), RAADSN (symptoms displayed now) (*β* = 0.26, *t* = 2.46, *p* < .05) and age (*β* = -0.06, *t* = -2.42, *p* < .05) predicted PDI. Sex (*β* = -0.54, *t* = -1.58, *p* = .12), education (*β* = -0.08, *t* = -1.28, *p* = .20), psychiatric diagnosis (*β* = 0.05, *t* = 0.14, *p* = 0.89 and Ritvo Autism Asperger Diagnostic Scale Revised symptoms displayed both when young and now (RAADSB) (*β* = 0.09, *t* =1.32, *p* = 0.19 did not predict PDI.

**PDI - CCQ components.** In the CCQ components model, Conspiracy (*β* = 0.05, *t* = 3.49, *p* < .001), Distrust (*β* = 0.09, *t* = 3.83, *p* < .001), ASRS (*β* = 0.12, *t* = 8.71, *p* < .001) RAADSY (*β* = 0.23, *t* = 2.39, *p* < .05) and RAADSN (*β* = 0.24, *t* = 2.28, *p* < .05) predicted PDI. Age (*β* = 0.05, *t* = -1.94, *p* = .053), sex (*β* = -0.34, *t* = 0.98, *p* = .33), education (*β* = -0.06, *t* = -0.91, *p* = .36), psychiatric diagnosis (*β* = 0.01, *t* = 0.02, *p* = 0.98, Fear/Action (*β* = 0.01, *t* =0.53, *p* = 0.59) and RAADSB (*β* = 0.11, *t* =1.51, *p* = 0.13) did not predict PDI.

**BADE – Total CCQ.** Examining the relationship between BADE and CCQ whilst controlling for the effect of ADHD and autistic traits, we found that PDI (N = 313, *β* = 0.03, *t* = 3.48, *p* < .001), CCQ Total (*β* = 0.01, *t* = 4.90, *p* < .001), RAADSB (*β* = -0.03, *t* = -2.27, *p* < .05), age (*β* = -0.02, *t* = -3.09, *p* < .01), sex (*β* = -1.55, *t* = 2.16, *p* < .05) and education (*β* = 0.04, *t* = 3.05, *p* < .01) predicted EII. We did find significant prediction of EII from psychiatric diagnosis (*β* = -0.1, *t* = -1.36, *p* = .17), ASRS (*β* = -0.06, *t* = -0.43, *p* = .67), RAADSN (*β* = -0.1, *t* = -0.52, *p* = .60) or RAADSY (*β* = -0.01, *t* = -0.32, *p* = .75).

**BADE – CCQ Components.** Including CCQ components instead of the total CCQ, we found that Conspiracy (N = 313, *β* = 0.2, *t* = 5.72, *p* < .001), age (*β* = -0.01, *t* = -2.57, *p* < .05), education (*β* = 0.04, *t* = 3.33, *p* < .001) and PDI (*β* = 0.02, *t* = 2.65, *p* < .05) predicted EII, while sex (*β* = -0.11, *t* = -1.54, *p* = .12), psychiatric diagnosis (*β* = -0.12, *t* = -1.72, *p* = .086) Fear/Action (*β* = -0.006, *t* = -1.87, *p* = .06), Distrust (*β* = -0.006, *t* = 1.18, *p* = .24), ASRS (*β* = -0.02, *t* = -0.13, *p* = .90), RAADSN (*β* = -0.02, *t* = -0.75, *p* = .45), RAADSY (*β* = -0.005, *t* = -0.26, *p* = .79) and RAADSB (*β* = -0.03, *t* = -1.93, *p* = .053) did not predict EII.

**Detailed results of the Path models**

**Path-models with total score of CCQ.** The first model using ML estimates showed that the regression of PDI on total score of CCQ was significant (*β* = 0.32, *z* = 6.11, *p* < .001), and that the regression of PDI on EII was significant (*β* = 0.18, *z* = 3.20, *p* = .001). The regression of EII on CCQ total was significant (*β* = 0.15, *z* = 2.86, *p* = .004). The model found that EII partially mediated the relationship between PDI and total CCQ (*β* = 0.03, *z* = 2.13, *p* = .033).

As our variables were non-normally distributed, we conducted a second analysis using WLS estimates and found that the regression of PDI on total score of CCQ was significant (*β* = 0.32, *z* = 6.75, *p* < .001), and that the regression of PDI on EII was significant (*β* = 0.18, *z* = 2.77, *p* = .006). Lastly, the regression of EII on CCQ total was significant (*β* = 0.15, *z* = 2.06, *p* = .040). The model did not, find that EII fully mediated the relationship between PDI and total CCQ (*β* = 0.03, *z* = 1.57, *p* = .117), when using the normalized data-set. However, since we had a specific hypothesis on the directionality of the relationships, EII nearly partially mediated the relationship between PDI and CCQ total (*p_one-tailed_* = .059) with a one-tailed test.

**Path-models with CCQ components.** The second path model using ML estimates showed that the regression of PDI on *Distrust* (*β* = 0.33, *z* = 6.21, *p* < .001), on *Conspiracy* (*β* = 0.28, *z* = 5.35, *p* < .001), and on EII (*β* = 0.18, *z* = 3.20, *p* = .001) while the regression of PDI on *Fear/Action* was not significant (*β* = -0.04, *z* = -0.69, *p* = .49). It also showed that the regression of EII on *Distrust* (*β* = 0.15, *z* = 2.79, *p* = .005) and on *Conspiracy* was significant (*β* = 0.24, *z* = 4.50, *p* < .001), while the regression of EII on *Fear/Action* was not significant (*β* = -0.11, *z* = -1.89, *p* = .058). The model showed that EII partially mediated the relationship between PDI and *Distrust* (*β* = 0.026, *z* = 2.10, *p* = .036) as well as between PDI and *Conspiracy* (*β* = 0.04, *z* = 2.61, *p* = .009), but not between PDI and *Fear/Action* (*β* = -0.02, *z* = -1.63, *p* = .103).

Using WLS estimates, we found that the regression of PDI on *Distrust* (*β* = 0.33, *z* = 6.52, *p* < .001), on *Conspiracy* (*β* = 0.28, *z* = 5.56, *p* < .001), and on EII (*β* = 0.18, *z* = 2.77, *p* = .006) was significant, while the regression of PDI on *Fear/Action* was not significant (*β* = -0.04, *z* = -0.68, *p* = .50). It also showed that the regression of EII on *Distrust* (*β* = 0.15, *z* = 2.48, *p* = .013) and on *Conspiracy* was significant (*β* = 0.24, *z* = 2.98, *p* = .003), while the regression of EII on *Fear/Action* was not significant (*β* = -0.11, *z* = -1.87, *p* = .062). The model found that EII did not fully mediate the relationship between PDI and *Distrust* (*β* = 0.026, *z* = 1.74, *p* = .083), between PDI and *Conspiracy* (*β* = 0.04, *z* = 1.85, *p* = .064), nor between PDI and *Fear/Action* (*β* = -0.02, *z* = -1.46, *p* = .15). However, as in the previous model, we had a specific hypothesis on the directionality of the relationships. Hence, EII partially mediates the relationship between PDI and Distrust (*p_one-tailed_* < .05), and between PDI and Conspiracy (*p_one-tailed_* < .05) with one-tailed tests.
